# In Vitro Bioaccessibility of Proteins and Bioactive Compounds of Wild and Cultivated Seaweeds from the Gulf of Saint Lawrence

**DOI:** 10.3390/md21020102

**Published:** 2023-01-31

**Authors:** Margarida M. M. Vasconcelos, Gabriela V. Marson, Laurie-Eve Rioux, Eric Tamigneaux, Sylvie L. Turgeon, Lucie Beaulieu

**Affiliations:** 1 Institut sur la Nutrition et les Aliments Fonctionnels (INAF), Département des Sciences des Aliments, Université Laval, Québec, QC G1V 0A6, Canada; 2 Fishing Engineering, Universidade Federal do Piauí (UFPI), Campus Universitário da Ininga, Teresina 64049-550, Brazil; 3 Merinov, École des pêches et de l’aquaculture du Québec (ÉPAQ), Cégep de la Gaspésie et des Iles, 6 rue du Parc, Grande-Rivière, QC G0C 1V0, Canada

**Keywords:** *Palmaria palmata*, *Saccharina latissima*, gastrointestinal model, protein release, disintegration, bioactivity

## Abstract

Despite the increased interest in macroalgae protein and fibers, little information is available on their bioaccessibility. The application of an in vitro gastrointestinal digestion model to study the degree of disintegration and release of proteins with expressed bioactivities from wild and cultivated *Palmaria palmata* and *Saccharina latissima* was proposed in this study. Macroalgae from the Gulf of St Lawrence, Canada, were submitted to digestive transit times of 2 (oral), 60 (gastric) and 120 (duodenal) minutes. Among wild samples, *P. palmata* had a higher percentage of disintegration, protein release and degree of hydrolysis than *S. latissima*. While the least digested sample, wild *S. latissima*, was the sample with the highest antioxidant activity (210 μmol TE g^−1^), the most digested sample, cultivated *P. palmata*, presented the highest ability to inhibit the angiotensin-converting enzyme (ACE), reaching 32.6 ± 1.2% at 3 mg mL^−1^. ACE inhibitory activity increased from 1 to 3 mg mL^−1^, but not at 5 mg mL^−1^. Wild samples from both species showed an ACE inhibition around 27.5%. Data suggested that the disintegration of the samples was influenced by their soluble and insoluble fiber contents. Further information on the bioaccessibility and bioactivity of these macroalgae should consider the characterization of digestion products other than protein, as well as the effects of previous product processing.

## 1. Introduction

A global interest in new and sustainable biomass to produce protein has resulted in attention towards the protein content of seaweeds [1]. Brown seaweeds are known to have a low protein content (3−15%, dry weight (d.w.)), while green seaweeds have a moderate content (9–26%, d.w.) and red seaweeds have the highest protein content at 47% (d.w.) [2,3]. At 35% (d.w.), *Palmaria palmata*, known as “dulse”, has protein levels, higher than those found in high-protein pulses, such as soybeans [4,5]. 

Despite their significant protein levels and the presence of numerous bioactive proteinaceous compounds in marine organisms [6], seaweeds are often used in human or animal food for their mineral content or for the functional properties of their polysaccharides [2]. Some edible seaweed proteins have better quality than those of higher terrestrial plants, being comparable in quantitative terms to legumes, with 30–40% (d.w.) in some species of red algae [7,8]. However, they have lower digestibility [8,9] due to the structure of their cell walls, which are rich in polysaccharides that form stable complexes with protein, rendering them inaccessible to proteolytic enzymes [10,11,12]. Among seaweeds, the red ones appear to have higher digestibility [11]. Thus, although macroalgae may be commonly used as food, the bioavailability of compounds from seaweeds in the human body is not sufficiently understood [10]. There is a scientific effort to elucidate macroalgae compounds’ bioaccessibility and bioavailability, due to the concern that macroalgae compounds, even when present in large amounts, may not be available after consumption and therefore will not be able to fully express their biological functions [12]. Bioavailability involves two different stages: bioaccessibility and bioactivity [13]. Bioaccessibility is the fraction of a compound released from its matrix in the gastrointestinal tract available for intestinal absorption [14]; bioactivity refers to the assimilation of the compound across intestinal cells, and the physiological response created due to its incorporation with the target site [13]. Thus, the whole bioavailability process involves digestion, absorption, transportation, utilization and elimination [15]. Although studies indicate that in vivo feeding methods, using animals or humans, usually provide the most accurate results, they are costly and time-consuming [10]. In vitro digestion models provide a useful alternative to in vivo models by rapidly screening food ingredients [16].

In this context, bioaccessibility studies are important to: (1) assess the true macroalgae quality and (2) evaluate the bioaccessibility of bioactive compounds and how these bioactivities are changed during and after the digestion process. Regarding the first point, the accurate consideration of a food as a source of protein is based on its amino acid composition and on the capacity of digestive enzymes to release [17], for the digestibility of these proteins is the primary factor in the availability of their amino acids [18]. Recent studies on macroalgae digestibility have been carried out to elucidate seaweeds protein bioacessibility and bioavailability [19]. The in vitro k-Protein Digestibility-Corrected Amino Acid Score (k-PDCAAS), which is considered an important parameter of protein quality, was highest for *P. palmata* (0.69 ± 0.01) among the seaweeds studied, indicating its potential as protein alternative [19]. Studies on in vitro algal protein digestibility are generally based on digestion using a single enzyme system (e.g., pepsin, pancreatin, etc.) [7,18,20], a multi-proteolytic enzyme system (e.g., trypsin, chymotrypsin, and peptidase/human intestinal juice, etc.) [8,21,22], or both methods [11,23]. Single-enzyme methods may be useful in predicting the digestibility of individual nutrients (e.g., protein, using pepsin). However, since digestion of a nutrient is generally influenced by the digestion of other nutrients, it is believed that multi-enzyme methods provide the most reliable values [24]. The protein nutritional value not only depends on the quality of protein, but also on the different components of food [25]. 

In addition to the assessment of the potential of macroalgae as alternative sources of protein, there is an increasing interest in understanding how the digestion affects bioactive compounds present in these matrices. For instance, *S. latissima* has been reported to have anti-inflammatory and antioxidant activities [26]. However, following in vitro digestion, a large decrease in the anti-inflammatory capacity was observed [26]. This finding reaffirms the need for bioacessibility studies, to understand how the bioactive compounds in seaweeds are modified during simulated digestion.

Besides variations in bioactivity observed following digestion, significant differences in seaweed bioactivities of different origins (geographic regions) and cultivation modes were observed [27]. Naturally, the protein composition of seaweeds may also vary depending on the species and other factors, such as the season, harvesting mode and environmental growth conditions [27,28,29,30]. The cultivation of selected seaweed species is a promising tool to control algae physiology and to standardize and enhance the production/activity of targeted bioactive compounds. For instance, high-nutrient cultivated *Ulva* spp. offer a sustainable source of fatty acids, pigments and phenolic compounds with attributable antioxidant and anti-inflammatory activities [31]. Tibbetts et al. [23] have studied the digestibility of cultivated red algae and reported that a temperate red seaweed (variant of *P. palmata*) may have value as a nutritional resource when cultivated solely for this purpose [22]. Seasonal variations of the protein content in seaweed have been demonstrated in studies [32] and are an important factor to consider. For instance, Galland-Irmouli et al. [18] demonstrated that the highest levels of proteins are found in *P. palmata* in the winter–spring period (21.9 ± 3.5%) and the lowest levels (11.9 ± 2.0%) occur in the summer–early autumn period, whereas most of the essential amino acids are present throughout the year [18]. Olischläger et al. [33] have shown that populations of *Saccharina latissima* harvested in the Arctic have a higher protein content at 4 °C compared to 10 °C [33].

Taking into consideration all of the above discussed aspects of macroalgae, recent works have studied the composition of several seaweeds [34], the influence of environmental and seasonal factors on them [32] and their potential as food ingredients [35]. Brown seaweed *S. latissima* and the red seaweed *P. palmata* are very relevant species in the Gulf of Saint Lawrence (Québec, QC, Canada) that represent a huge potential as bioactive food ingredients. We have shown, in previous studies, that these species possess interesting bioactive peptides (antioxidant, antihypertensive and antimicrobial) [36,37]. Soluble seaweed extracts of both *P. palmata* or *S. latissima* with antioxidant capacity (ORAC) and angiotensin-converting enzyme (ACE)-inhibitory activities were also studied as additives to Camembert-type cheese [38]. The influence of environmental conditions and cultivation mode (wild or cultivated) have been demonstrated in the composition and bioactive properties of these seaweeds [27,32]. Thus, previous research demonstrates the interesting bioactive profiles of *S. latissima* and *P. palmata*, but their bioactivity following digestion is still unknown. The objective of the present study was to evaluate the protein digestibility of *S. latissima* and *P. palmata* of the Gulf of Saint Lawrence, in the Boreal region. We proposed the application of a gastrointestinal digestion model to better understand macroalgae disintegration, protein release and the bioactive profile (antioxidant and ACE-inhibitory activities) before and after digestion of samples from different species and cultivation modes (wild and cultivated). To our knowledge, no study has been conducted on the rate of disintegration of macroalgae in the gastric environment under the conditions applied in this research.

## 2. Results

Dried wild and cultivated samples of *P. palmata* and *S. latissima*, before the digestive process, had fiber (soluble and insoluble) and protein contents as indicated in Table 1. Overall, *P. palmata* has a 2-times higher soluble fiber content and 1.25-times higher protein content than *S. latissima*. Between wild and cultivated *P. palmata*, the latter presents a higher insoluble fiber than the other; otherwise, the composition of the two is very similar. Wild *S. latissima* samples present a higher soluble and insoluble fiber content than cultivated samples from the same species.

### 2.1. Water Holding Capacity of Seaweeds

**Figure 1:**
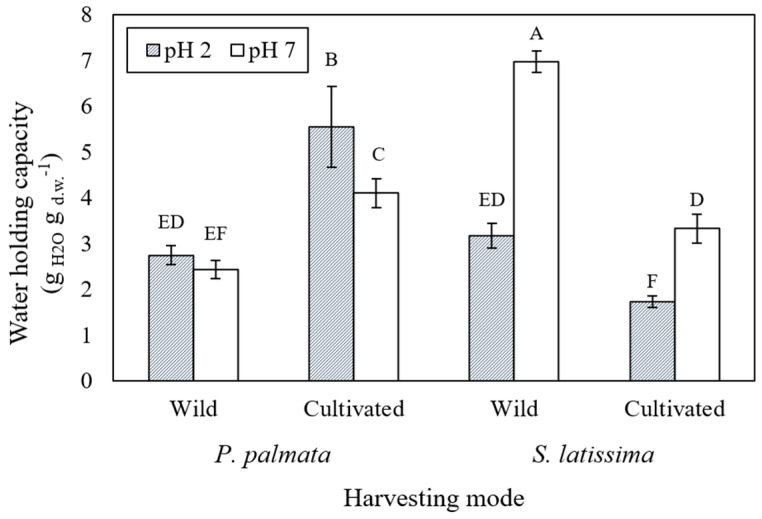
Evaluation of the water holding capacity (WHC) of the cultivated and wild *P. palmata* and *S. latissima* samples as a function of pH. Values are presented as mean ± standard deviation (*n* = 3). Histogram bars with different letters are statistically different according to Tukey’s Studentized Range (HSD) at *p* < 0.05, comparing all conditions. Error bars represent standard deviations.

The evolution of the water holding capacity (WHC) of *P. palmata* and *S. latissima* (wild and cultivated samples) at pH 7.0 and 2.0 is shown in Figure 1. According to the general linear mixed model (GLMM), the WHC of the cultivated *P. palmata* sample increased (from 4.10 ± 0.32 to 5.55 ± 0.88 g_H2O_ g_d.w._^−1^) when the pH decreased from 7 to 2. Converse behavior was observed in both samples of *S. latissima*, with a decrease in WHC (6.97 ± 0.24 to 3.17 ± 0.28 and 3.33 ± 0.32 to 1.73 ± 0.13 g_H2O_ g_d.w._^−1^) with decreasing pH from 7.0 to 2.0, to wild and cultivated, respectively. The highest WHC was measured in the wild *S. latissima* sample (6.97 ± 0.24 g_H2O_ g_d.w._^−1^) at pH 7.0, and the lowest was measured in both the cultivated *S. latissima* sample at pH 2.0 (1.73 ± 0.13 g_H2O_ g_d.w._^−1^) and wild *P. palmata* at pH 7.0 (2.43 + 0.20 g H_2_O g_d.w._^−1^) (Figure 1).

### 2.2. In Vitro Digestion Model

**Figure 2:**
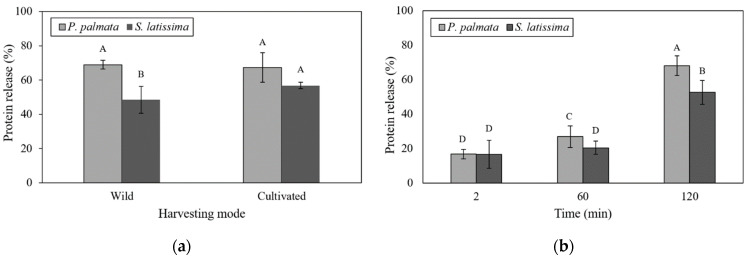
(**a**) Protein release as a function of harvesting mode of wild and cultivated *P. palmata* and *S. latissima* samples after 120 min of in vitro gastrointestinal digestion; and (**b**) protein release of *P. palmata* and *S. latissima* samples (mean of both wild and cultivated) for each digestive stage. Histogram bars with different letters are statistically different (*p* < 0.05). Statistical analysis for each graph was performed, comparing different species and treatments (harvesting mode or digestion period). Error bars represent standard deviations (*n* = 3).

The digestion process used dried seaweed samples with a particle size of 0.5 mm and at a concentration of 0.5 g mL^−1^. Physiological transit times (2, 60 and 120 min) represented the digestive stages of G0 (gastric digestion at time zero), G60 (gastric) and D120 (duodenal). At the end of the digestive process (D120), all samples were viscous, except for the wild *S. latissima* sample (data not shown). Figure 2a,b present the protein release observed in the two macroalgae regarding the harvesting mode and digestion time, respectively.

As shown in Figure 2a, wild *S. latissima* had a significantly lower percentage of protein release than other treatments (25.74 ± 2.01%). In *P. palmata*, the release of this nutrient was not significantly affected by the interaction of the species with their respective harvesting modes (wild and cultivated).

**Figure 3:**
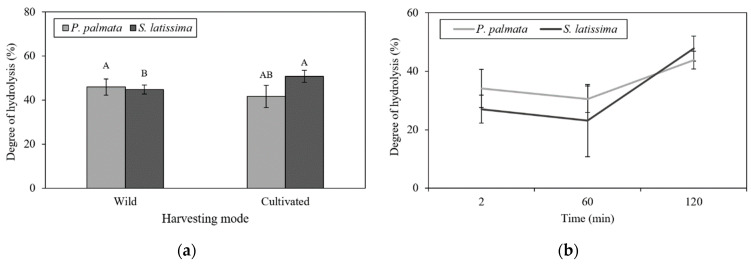
(**a**) Degree of hydrolysis as a function of harvesting mode of wild and cultivated *P. palmata* and *S. latissima* samples after 120 min of in vitro gastrointestinal digestion; and (**b**) degree of hydrolysis of *P. palmata* and *S. latissima* samples (mean of both wild and cultivated) for each digestive stage. Histogram bars with different letters are statistically different (*p* < 0.05). Statistical analysis for each graph was performed, comparing different species and treatments (harvesting mode or digestion period). Error bars represent standard deviations (*n* = 3).

Figure 3a,b present the degree of hydrolysis of wild and cultivated *P. palmata* and *S. latissima*, regarding the harvesting mode and digestion time, respectively. Corroborating protein release results, the release of amino acid residues (DH) followed the same tendency (Figure 3a), with no difference detected between cultivated *P. palmata* and *S. latissima*. However, among wild samples, *P. palmata* presented a higher protein release and DH (Figure 2a and Figure 3a) compared to *S. latissima* samples.

Protein release showed a strong interaction with species and digestion times (Figure 2b). *P. palmata* had the highest percentage of protein release in the duodenal (D120) stage (68.16 ± 2.61%). This release increased significantly over time during the digestive process for this species. However, at the end of the digestive process, the proteins achieved a DH of only 43.79% (Figure 3b). In addition, despite the low protein release in G0 and G60 stages, as shown in Figure 2b, the DH was greater than 30% (Figure 3b).

For *S. latissima* samples, this increase in protein release was only observed at 120 min of duodenal digestion (52.68 ± 2.61%). This result was significantly lower than that found in *P. palmata* samples at this same digestion time (Figure 2b). In addition, the G0 and G60 stages were not significantly different in protein release for *S. latissima* samples. DH values followed a similar trend, as shown in Figure 3b, with levels below 30%. The greatest release of amino groups was at the D120 stage; however, no significant difference between *P. palmata* (43.79%) and *S. latissima* (41.34%) was observed at this stage (Figure 3b). 

### 2.3. Undigested Nutrients 

**Figure 4:**
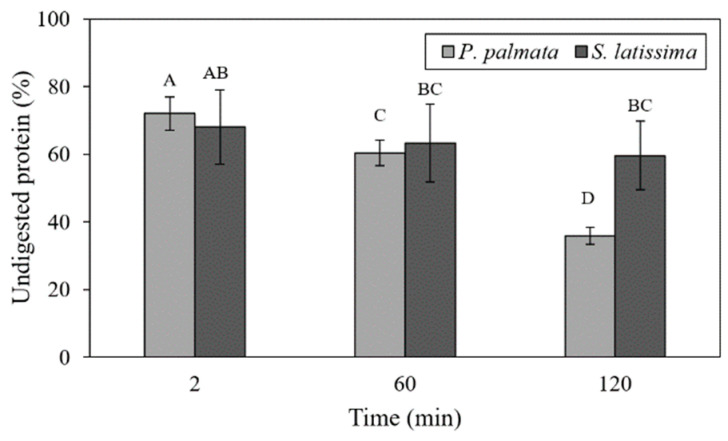
Effects of various digestion stages on the percentage of undigested proteins during *in vitro* gastrointestinal digestion of wild and cultivated *P. palmata* and *S. latissima* samples. Histogram bars with different letters are statistically different (*p* < 0.05). Statistical analysis was performed, comparing different species and digestion periods. Error bars represent standard deviations (*n* = 3).

Figure 4 shows comparative histograms of undigested proteins (UP), which are strongly affected by the seaweed digestion time. *P. palmata* samples had the highest UP content at the G0 stage (72.06%), similar to *S. latissima* at the G0 stage. At the three stages of digestion, significant reductions in UP content were observed, with the lowest content occurring at D120 (35.93%). However, *S. latissima* samples were not affected by duodenal digestion (D120), with a small reduction in UP found only for gastric digestion (63.32%). Undigested proteins at the D120 (r = 0.77; *p* < 0.01) and G60 (r = 0.65; *p* < 0.05) digestive stages were positively correlated with WHC at pH 7.0.

**Figure 5:**
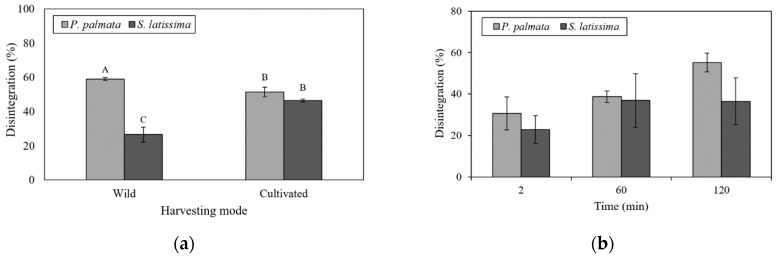
(**a**) Disintegration percentage of wild and cultivated *P. palmata* and *S. latissima* samples after 120 min of in vitro gastrointestinal digestion; and (**b**) disintegration percentage of *P. palmata* and *S. latissima* samples (mean of both wild and cultivated) per digestive stage. Histogram bars with different letters are statistically different (*p* < 0.05). Statistical analysis for each graph was performed, comparing different species and treatments (harvesting mode or digestion period). Error bars represent standard deviations (*n* = 3).

Significant interaction of seaweed species and harvesting mode were observed regarding the disintegration percentage (Figure 5a). Wild samples of *P. palmata* and *S. latissima* presented the highest (45.60 ± 0.85%) and lowest (25.38 ± 1.83%) disintegration percentage, respectively, but no significant differences were observed between cultivated species.

Both species showed low levels of disintegration during G0 (2 min), with *S. latissima* being significantly lower (22.89 ± 2.21%) (Figure 5b). Samples of *P. palmata* presented increasing disintegration with digestion time, reaching a maximum after 120 minutes of duodenal digestion (55.22 ± 0.96%) (Figure 5b). The disintegration percentage of *S. latissima* samples were not significantly affected by duodenal digestion (D120) (Figure 5b). However, strong inverse relationships were found between disintegration and UP at the G60 stage (r = −0.89; *p* < 0.0001) and the D120 stage (r = −0.95; *p* < 0.0001). Insoluble fiber correlated positively with UP in G60 (r = 0.74; *p* < 0.01) digestion stages. Negative correlations were also found between disintegration and WHC at pH 7.0 at both G60 (r = −0.79; *p* < 0.01) and D120 stages (r = −0.92; *p* < 0.0001). A strong negative correlation between insoluble fiber and soluble fiber (r = −0.98; *p* < 0.0001) and insoluble fiber and disintegration in G0 (r = −0.87; *p* < 0.001) was found. Soluble fiber was strongly associated with disintegration in the gastric digestion stage (r = 0.83; *p* < 0.001). 

Insoluble fiber correlated positively with UP in the G60 (*r =* 0.74; *p <* 0.01) digestion stage. In contrast, soluble fiber was negatively correlated with UP at G60 (*r* = −0.65; *p* < 0.05), and UP at G0 (*r* = −0.64; *p* < 0.05) stages. 

However, undigested nutrients, which comprise all nutrients not digested after the in vitro model, such as lipids and ashes, were also associated with the disintegration percentage in the G60 stage (*r* = 0.79; *p* < 0.01). In this study, no relationship was found between soluble fiber, insoluble fiber and undigested nutrients and the disintegration percentage in the duodenal digestion stage.

### 2.4. Bioactivities

**Figure 6:**
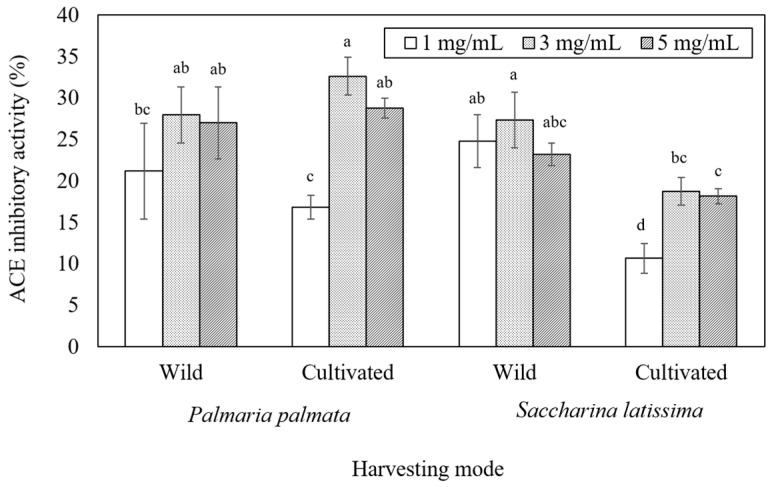
ACE inhibitory activity (%) of *P. palmata* and *S. latissima* samples resulting from the duodenal digestion supernatant (D120). Statistical analysis was performed, comparing the mode of cultivation and sample concentration for each species separately. Error bars represent standard deviations (*n* = 3).

ACE inhibitory activity results are presented in Figure 6. ACE inhibitory activity increased from 1 to 3 mg mL^−1^, but no difference or a decreased inhibition was observed at 5 mg mL^−1^ when compared to 3 mg mL^−1^. Highest inhibition among samples was observed at 3 mg mL^−1^ for cultivated *P. palmata* (Figure 6). No significant differences were found in the ACE inhibition between wild *P. palmata* (28.0 ± 4.3%) and *S. latissima* (27.3 ± 1.4%) at the highest observed concentration evaluated (3 mg mL^−1^) (Figure 6). However, for cultivated samples, *P. palmata* presented a higher inhibitor level, reaching 32.6 ± 1.2% at 3 mg mL^−1^ against 18.8 ± 0.9% for *S. latissima* at the same sample concentration. When comparing wild versus cultivated samples, although *P. palmata* did not show significant differences, a clear difference among *S. latissima* was observed for all concentrations tested with higher ACE inhibition observed for wild samples (Figure 6).

Regarding the antioxidant activity of samples, wild *S. latissima* duodenal supernatant had the highest observed ORAC value (210 ± 7.18 μmol TE g^−1^). Regardless of the harvesting mode, ORAC values for *P. palmata* samples were too low to be detected. In addition, the antioxidant capacity (99.62 ± 8.29 µmol TE g^−1^) of cultivated *S. latissima* was significantly lower than the other sample.

## 3. Discussion

### 3.1. Sample Characterization

Water holding capacity (WHC) is a parameter that measures the amount of water that the material is capable of absorbing. In macroalgae, this parameter is related to the composition of the cell walls, the type and amount of polysaccharides, and some cell wall proteins present in the structure [39]. Water retention measurements are related to texture properties of food and are very relevant for meat substitutes and alternative sources of protein [40]. A distinct pH-related behavior regarding WHC was observed in *P. palmata* and *S. latissima*. WHC increased as pH decreased in the cultivated *P. palmata* samples, while the opposite trend was observed in *S. latissima* samples. The *S. latissima* results agree with Jiménez-Escrig and Sánchez-Muniz [41], since gastric pH levels decreased the WHC in this species. The same trend was observed in another study that reported a decrease in WHC due to lowering of pH in brown seaweed samples, while WHC increased in red seaweed samples [42]. Conversely, a study by Lahaye et al. [43] has indicated that the WHC of *P. palmata* species was not markedly affected by pH. 

Differences in WHC between the two species may be related to their differing chemical composition, since this physicochemical property has been used to refer to the ability of dietary fiber to hold water under specific conditions [42]. Preliminary studies showed low levels of soluble and high levels of insoluble fibers in *S. latissima* samples, with the opposite found in *P. palmata* (Table 1). WHC is related to the characteristics of seaweed polysaccharides and the different protein conformations in the cell wall. The WHC can contribute not only to the hydrophilic fraction of proteins and polysaccharide molecules, but also to capillary action in their three-dimensional structures [44,45,46]. The differences in WHC between the two species can possibly be attributed to these chemical characteristics.

### 3.2. In Vitro Digestion Model

The rate of protein release was not affected by the difference in species or its harvesting mode, except for wild *S. latissima* (Figure 2a). This effect is probably due to the similar soluble fiber content in *P. palmata* species, whatever the growth conditions, and to the low level of soluble fiber in cultivated *S. latissima*. As presented in Table 1, wild and cultivated *P. palmata* samples had high levels of fiber, notably soluble fiber (10.52–10.61%, respectively), compared to *S. latissima* (5.91% for wild and 3.77% for cultivated). However, the synergistic and antagonistic behavior of seaweed compounds might significantly affect the utilization of individual nutritional factors [10]. Thus, protein release may be influenced by the presence of other nutrients. Studies simulating gastrointestinal conditions have indicated that, rather than having discrete xylan solubilization in different compartments of the digestive tract, soluble fibers tend to be continuously extracted along the tract as digestion proceeds [41]. Other research indicates that alginates present in brown algae are probably solubilized preferentially during the intestinal phase [46]. Generally, protein digestibility can differ according to the species, seasonal variations, and the presence of other compounds, such as phenolics, polysaccharides and color compounds [17], which are different among red and brown macroalgae. It is well known that the level of phenolic compounds is lower in *P. palmata* than in *S. latissima* [23] and could explain why the protein release efficiency was higher in *P. palmata* than in *S. latissima*. In brown algae, various amounts of polyphenols and other compounds are usually associated with Klason lignin that may be responsible for their higher insoluble fiber content [47]. Lignin, as a component of dietary fiber, may also decrease the seaweed protein digestibility, because its phenol units can be complexed with proteins, and this fact could explain the lower digestibility of vegetable proteins [25].

Protein release during different digestion times (G0, G60 and D120) was significantly different, depending on the seaweed species (Figure 2a). Protein release in *P. palmata* samples increased significantly from G0 (16.79%) to D120 (68.16%); however, in *S. latissima* samples, this increase was only observed from stage G60 to D120 (20.80% to 52.68%). For *S. latissima*, this result was strongly influenced by the release of protein in the cultivated samples (Figure 2b). In vitro digestibility of different edible algae products showed that the highest digestibility values were observed after enzymatic hydrolysis of red seaweed, while brown seaweed produced the lowest digestibility values [24]. Machů et al. [24] state that the higher digestibility values of red seaweeds could be caused by a higher content of soluble dietary fiber. Polysaccharides, one the main compounds in seaweeds, play a role in their digestibility. Brown seaweeds have mostly alginate, fucoidan and laminarin in their intercellular matrix and mannitol as a storage carbohydrate [48,49], while in red seaweeds, such as *P. palmata*, the main polysaccharides are agar, carrageenan and xylans [48,50]. Firstly, the solubility of those polysaccharides is different, with agar being the least soluble, followed by carrageenan and then alginate and sulfated fulcans [48]. For instance, as alginate in brown seaweed is more viscous than the other polysaccharides, this high viscosity decreased the rate of diffusion of the enzymes and nutrients and, consequently, the rate of digestion and absorption [51]. Under the conditions used in this study, it can be suggested that the rheological behavior of the polysaccharides as a function of the chemical conditions of the medium have a decisive effect on the protein release. This happens because a higher concentration of soluble fiber in the enzymatic system results in greater viscosity and pepsin activity inhibition [52].

In this work, protein release increased after duodenal digestion (pancreatin for 120 min after deactivation of pepsin). Studies that applied the same gastrointestinal model using a 50% reduction in the number of digestive enzymes indicated that the release of amino acids was greater in heat-treated samples than in unheated ones [53]. However, Marrion et al. [54], evaluating the improvement of in vitro digestibility by means of physical processes, indicated that digestibility was better for unheated samples than for heated samples, and for non-ground vs. ground samples, showing that grinding in liquid nitrogen led to better digestibility. Dried *P. palmata* and *S. latissima* samples exposed to a standardized mixture of commercial purified enzymes produced 26.7% and 6.6% of digestible proteins, respectively [23]. According to Tibbetts et al. [23], high variability in the results of in vitro assays is common and may be related to species differences but is more likely due to distinctions in the methods used (the enzyme mixtures, the extent of sample processing and the assay conditions). Based on these facts, despite methodological particularities, in the present research, the results of protein release from *P. palmata* (68.15%) and *S. latissima* (52.68%) were higher than those reported in the literature. However, the statistical data showed no differences regarding seaweed harvesting mode and the observed digestive times. Thus, regardless of the harvesting mode, *P. palmata* and *S. latissima* species showed significant protein release using the in vitro digestion model. 

The level of protein degradation caused by proteolytic enzymes simulating human gastrointestinal digestion was estimated by the DH. As expected, the highest rate of protein degradation was reported at the end of the duodenal digestion (Figure 2b). The highest percentage of released amino groups was observed in the cultivated *S. latissima* sample. This relationship was weaker in *P. palmata*. It is known that the specificity of the enzymes used for hydrolysis may influence the size and number of peptides formed and amino acid groups released [36]. Previous studies indicated that the association of BSA with a protein extract from *P. palmata* produced a significant decrease in the rate of hydrolysis with trypsin, chymotrypsin and intestinal juice [18]. Thus, in this research, the inclusion of BSA in both gastric and duodenal juices may have influenced the results. However, regardless of the type of enzymes used, *P. palmata* proteins are generally hydrolyzed only to a limited extent [18].

The presence of soluble carbohydrates and their interactions with proteins or proteolytic enzymes can reduce protein hydrolysis [55]. This phenomenon happens because soluble fibers form a dispersion when mixed with water and can increase the viscosity of the medium, delaying the diffusion of digestive enzymes and the consequent absorption of nutrients [56]. Soluble fiber fractions of *P. palmata* consist of linear β−1.4/β−1.3 mixed linked xylans, containing similar numbers of 1,4 bonds, while insoluble fibers are formed essentially of xylan with some 1,3 linked xylose and a small amount of cellulose [43]. In addition, *S. latissima* is rich in insoluble fiber (13.92% to 32.44%) (Table 1), mostly cellulose (β-l,4-D-glucan), which is insoluble in dilute base or acid [57]. These factors together (type and enzymatic action), as well as the nature of the polysaccharides, could be determinants in the protein hydrolysis of *P. palmata* and *S. latissima*.

### 3.3. Undigested Nutrients 

Indigestible fraction values are considered more physiologically relevant than dietary fiber values as an accurate way to analyze enzymatic digestion [58,59]. An inverse association (Pearson’s correlations) between the disintegration extent of samples and the amount of undigested protein left after all digestion stages indicate that the smaller the disintegration is, the higher the protein content found in the undigested material. Another interesting association was observed between disintegration and the insoluble fiber content, suggesting that samples with more insoluble fiber disintegrated less. Inverse correlations between disintegration and WHC before digestion may be related to this content of insoluble fiber, which limits the capacity of samples of holding water, and then also limits their disintegration. Finally, the amount of undigested nutrients correlates well with the disintegration of samples. In this study, *S. latissima* samples were partially digested at the end of the duodenal stage (59.61% undigested protein), while *P. palmata* samples had 35.93% undigested proteins at this stage. The fact that pepsin-resistant protein is lower in red seaweeds than in brown seaweeds [58] may explain the differences in the digestive process in both species. Indeed, low protein digestibility also is reported for macroalgae in in vivo studies [9,60]. Macroalgae pre-treatments, such as fermentation and heat treatments, have been evaluated, aiming to increase digestibility [9,53] and enhance bioactivity [61]. A higher release of amino acids is often reported, but an increased nitrogen digestibility is not always seen [9,53].

Protein structure may also be significantly affected by the physico-chemical conditions found in the gastrointestinal tract, affecting the rate of proteolysis [62]. Based on this data, using intervals for the gastric pH between pH 2.0 and 3.0 after the addition of digestive juices [63] may favor insolubility and precipitation of alginates [64]. *S. latissima* samples were collected during summer, a time when this species accumulates alginates and other soluble carbohydrates, such as laminaran and mannitol [65]. Laminarans can be solubilized at pH 2.0, whereas alginate is solubilized at pH 7.5, at 35 °C [66]. Residual proteins also appear to have affected digestion. Samples’ WHC at pH 7.0 was positively correlated with the content of UP at G60 and D120 digestive stages and negatively correlated with disintegration, in both stages, suggesting that the remaining undigested proteins contributed to the capacity of samples to retain water.

The most important function of the stomach is mixing and disintegrating the meal into a semi-solid mixture that can be continuously delivered into the intestine for further digestion and absorption [67]. Disintegration indicates how fast a food particle can break into small fragments so that entrapped nutrient ingredients can dissolve into the gastric juice [68]. The dissolution and absorption numbers are governed by properties such as concentration, particle size, intestinal permeability, and residence time [69].

In this study, disintegration was strongly influenced by the seaweed harvesting mode (wild and culture) and the digestion times (2, 60 and 120 min). For both species, the best disintegration results obtained were 55.22% (*P. palmata*, duodenal digestion stage) and 36.96% (*S. latissima* gastric digestion stage). In vivo studies indicate that repeated shearing and grinding of gastric contents by the peristaltic movements of the stomach wall, together with the biochemical degradation caused by acids and enzymes, facilitate the progressive digestion of solid food until it is suitable for further digestion in the intestine [67]. This may explain why samples with low disintegration rates during gastric digestion also had low rates of disintegration in duodenal digestion.

Strong negative correlations between seaweed disintegration extent and undigested protein content for both G60 and D120 stages confirm the effect of the gastrointestinal digestion process in macroalgae protein. Moreover, disintegration during gastric digestion (G60) was positively correlated with soluble fiber content and negatively correlated with the insoluble fiber content, indicating that the extent of disintegration at the gastric stage was higher in samples with a higher soluble fiber content and smaller insoluble fiber content. Additionally, the higher the insoluble fiber content in samples, the higher the amount of undigested proteins was, especially in the gastric digestion stage (G60), while an opposite relationship was found for soluble fiber content. High rates of disintegration are indeed associated with a weak cell structure [70]. These data suggest that the rigid cell structure of the macroalgae studied, especially represented by the insoluble fiber content, was a key determinant in the disintegration of *P. palmata* and *S. latissima* samples in the gastric digestion stage. These results seem to be the first reported on the rate of disintegration of macroalgae and the relation to macroalgae composition in the gastric environment under the conditions applied.

### 3.4. Bioactivity

Bioactivity results showed that seaweed samples were able to inhibit ACE to a maximum of 33% at a concentration of 3 mg mL^−1^, and *S. latissima* samples presented antioxidant activity. Cultivated *P. palmata* had the highest ACE inhibition level, while wild *S. latissima* had the highest antioxidant capacity. The later sample had the lowest degree of protein release. Macroalgae samples have a complex composition, and it can be inferred that other compounds, such as antioxidants, including phenolic compounds, carotenoids, fucoxanthin, and isoprenoids [71], apart from proteins and peptides, may also be involved in ACE inhibition and antioxidant activity. 

A sequence of digestive enzymes (amylase (A3176), pepsin, lipase and pancreatin) was used in this study. Studies based on *P. palmata* digestion using a unique enzymatic system (chymotrypsin) showed that, when evaluating the antioxidant activity by the ORAC assay, hydrolyzed fractions (molecular weight < 10 kDa) of cultivated samples presented higher activity (440.73 ± 43.21 μmol TE g^−1^) [4] when compared to wild ones (261.93 ± 19.41 μmol TE g^−1^) [36]. Considering the methodological differences, the results in this work were comparatively smaller for *P. palmata* samples (too low, data not shown) and comparable for wild *S. latissima* samples. However, in the present research, the multienzyme system used certainly contributed to the release of other chemical compounds besides proteins, which may have interfered with the results. 

Other studies have shown that the pepsin digest of *Pyropia yezoensis* (previously known as *Porphyra yezoensis*) had the most potent ACE inhibitory activity among digests tested with protease P, denazyme AP and bioprase PN4 [72]. Furthermore, enzymatic hydrolysis by chymotrypsin enhanced both the antioxidant and ACE inhibitory activities of protein extracts of wild and cultivated *P. palmata* [27,36]. Tests on the action of *Undaria pinnatifida* (wakame) on blood pressure and other metabolic disorders in hypertensive patients have shown the presence of ACE inhibitory peptides in a peptic digest, which may be responsible for the observed blood pressure reduction in these patients [73]. Digested phycoerythrin apoprotein released a phycoerythrobilin compound during mammalian gastrointestinal digestion, and the apoprotein’s antioxidant capacity was greatly enhanced by the phycoerythrobilin compound compared to *Porphyra* spp. extracts [20]. Bondu et al. [36] reported an antioxidant activity for protein hydrolysates of *S. longicruris* (<250 µmol of Trolox equivalents (TE) g^−1^) and an ACE (IC_50_ of 460.05 mg mL^−1^) for < 10 kDa fraction of *P. palmata* hydrolyzed with chymotrypsin. An anti-inflammatory activity of photosynthesis-related compounds in *P. palmata* extracted in water has been studied [61]. Antioxidant properties (ORAC 35.8 µmol TE g^−1^) of a *P. palmata* extract, produced using a combined enzymatic and water extraction procedure, was reported by Wang et al. (2010) [74].

Research with peptides extracted from microalgae production waste suggested that the antioxidant activity of purified peptides could be preserved after treatment with pepsin and pancreatin enzymes [70]. However, low antioxidant activity and the loss of anti-inflammatory activity was reported for *S. latissima* aqueous or ethanolic extracts [26]. Digestion with pepsin and pancreatin releases phenolic compounds linked to proteins [75], and extreme pH changes release bound phenolic compounds from cell wall polysaccharides, thus making them available for measurement [76]. Phlorotannins are phenolic compounds that can form strong complexes with proteins, either reversibly by hydrogen bonding through peptide or amide linkages, or irreversibly by covalent condensation [75]. In addition, a positive correlation between the phenolic compounds and antioxidant activities of seaweed extracts has been reported [71]. Other studies have also shown that, in brown seaweeds, enzymatic extraction allowed the extraction of phlorotannins and carbohydrate compounds with ACE inhibitory activity [76]. In the present research, despite having an important protein content before the digestive process (Table 1), the wild-type *S. latissima* sample presented the lowest values of protein release and DH when submitted to in vitro gastrointestinal digestion. This sample was the one with the best antioxidant activity and relatively high ACE inhibitory activity. Thus, it can be inferred that the probable presence of phenolic compounds may have interfered with the antioxidant and ACE inhibitory activities of the *S. latissima* samples.

## 4. Materials and Methods

### 4.1. Chemicals

The reagents used to prepare the digestion solution, NaCl (S271-3), NaHCO_3_ (80500-300), CaCl_2_ (C614-3), and HCl (UN 1789), were purchased from Thermo Fisher Scientific (Hampton, VA, USA). The KCl (3040) and KH_2_PO_4_: monobasic crystal (4008-01) were acquired from J. T. Baker (Phillipsburg, NJ, USA), and KSCN (CAAA14318-22) and NH_4_Cl (CAAA12361-36) were supplied by VWR (Radnor, PA, USA). The NaH_2_PO_4_·H_2_O (84456-300), Na_2_SO_4_ (57361) and MgCl_2_.H_2_O (M-2670) were purchased from Anachemia, BDH and Sigma-Aldrich (Oakville, ON, Canada), respectively. The α-amylase (A3176), pepsin (P7000), lipase (L3126), bile (B8631), mucin from porcine stomach (Type III), pancreatin (P7545), angiotensin-converting enzyme from rabbit lungs (EC 3.4.15.1), its substrate N-hippuryl-His-Leu (HHL), as well as 1,4-dioxane, were supplied by Sigma-Aldrich (Oakville, ON, Canada). Bovine serum albumin (BSA) was acquired from Thermo Fisher Scientific (Hampton, VA, USA). The specific ACE inhibitor, enalapril, was supplied by Sigma-Aldrich (St. Louis, MO, USA). The α-amylase (E-BLAAM), protease (E-BSPRT), amyloglucosidase (E-AMGDF) and celite (G-CEL 100) were purchased from Megazyme International Ireland Ltd. (Wicklow, Ireland). 

### 4.2. Sample Characterization

Wild samples of *P. palmata* (Linnaeus) (F. Weber & D. Mohr, 1805) and *S. latissima* (Linnaeus) (C.E. Lane, C. Mayes, Druehl & G.W. Saunders) were harvested from the coastal areas of the Rivière-au-Renard and Lower Saint Lawrence (Québec, QC, Canada), respectively, in June 2015. Samples of *P. palmata* were grown in tumble culture, in indoor tanks at École des pêches et de l’aquaculture du Québec (ÉPAQ) (at Merinov Research Station) between February and April 2016. Young (≤1-year) *P. palmata* fronds were cultivated in several 140 L plastic tanks (0.55 m (L) × 0.55 m (L) × 0.45 cm (h); 0.3 m^2^ surface area). *S. latissima* (Sugar Kelp) was cultivated on submerged longlines of a marine farm in Îles-de-la-Madeleine (Québec, QC, Canada) and harvested in August. 

Seaweed samples were classified, washed in filtered seawater, and gently scraped to remove their epibionts. Next, macroalgae were dried for 48 h in an oven (model: HeraTherm OGS400, Thermo Fisher Scientific, Montreal, QC, Canada) at a temperature of 40 °C until their water content was ≤20%. Dried seaweeds were reduced into flakes using a cutter/mixer (model: UMC 5 electronic, Stephan, Hameln, Germany). Seaweed flakes were subjected to fine grinding (model: Thermomix, Vorwerk, Wuppertal, Germany) and finally stored in vacuum-sealed bags until use. Approximately 550 g of dried seaweed samples were placed into a mixer-mortar-grinder (RM 100, Retsch GmbH, Haan, Germany), frozen in liquid nitrogen and ground. This procedure was performed with both wild and cultivated seaweed samples. The resulting dried seaweed was collected in plastic bags, weighed, and stored at 4 °C until use. 

Dried wild and cultivated samples of *P. palmata* and *S. latissima*, before the digestive process, were characterized according to their fiber (soluble and insoluble) and protein contents (Table 1). Protein content was determined by the Kjeldahl method, as described by Vasconcelos et al. [32]. The insoluble and soluble fiber contents of dried seaweeds were measured following AOAC Method 991.43 [77] using the Megazyme Kit (Bray, Ireland).

Water holding capacity (WHC) analysis of dried seaweed samples was performed by the centrifugation method described by Suzuki et al. [42]. The WHC of dried seaweed was expressed as the weight in grams of water held by 1 g of the sample (d.w.) [22]. At the same time, the samples were immersed for 30 min in water adjusted to pH 2.0, with hydrochloric acid to simulate gastric pH conditions [68]. All analyses were performed in triplicate.

### 4.3. In Vitro Digestion Model

Before initiating the digestive processes, the particle size of dried seaweed samples was standardized, because particle size could affect the availability of nutrients for digestion [78]. The sample was placed in a top sieve and tapped. As a result, larger particles were retained, while smaller particles passed through. At the end of sieving, powdered samples with 32−100 mesh (0.5 mm) were obtained. 

In the model used, the digestion process of the human gastrointestinal tract was simulated in a simplified manner by applying physiologically-based conditions, such as pH and residence time periods, typical for each compartment [63]. The large intestine was not considered, because food digestion and absorption mainly take place in the small intestine, in vivo [79]. Based on this, in vitro digestion models described by Oomen et al. [79] and Versantvoort et al. [63] were used, with some modifications [63,79]. Preliminary tests showed (data not shown) that using 3 g of seaweed in 6 g of water with transit times of 2 min for oral digestion, 60 min for gastric (G60) and 120 min for duodenal digestion (D120) provided the best release of soluble proteins in the in vitro digestive process. The proportion of digestive juices, based on human physiology, was determined according to the criteria described by Versantvoort et al. [63]. As a result, a mass ratio of 1:2:2:1 (saliva: gastric juice:duodenal juice:bile) was used. The composition of artificial juices (saliva, gastric, duodenal and bile) added at each digestion step was previously described by Versantvoort et al. [63].

The enzymes were added to the digestive juices, and the process was started by adding 3 g of seaweed to 6 mL of water in a beaker (400 mL) and incubating in a hot bath (37 °C) for 2 min with overhead mixing (75 rpm). Next, saliva juice (4 mL saliva, 1 mL mucin, and 1 mL α-amylase (A3176)) was added and mixed for another 2 min, simulating mastication. Samples were then placed in a shaking bath (37 °C; 200 rpm); gastric juice (5 mL gastric solution, 4 mL mucin + BSA and 3 mL pepsin) was added; and the mixture was shaken for 1 h. Finally, duodenal juice (8.9 mL duodenal solution, 0.5 mL BSA, 1 mL lipase and 3.6 mL pancreatin) and bile juice (6 mL) were added and mixed for 2 h (37 °C; 200 rpm).

After adding saliva juice to *S. latissima* samples, low hydration capacity was observed, compromising the efficiency of the subsequent centrifugation. For this reason, gastric juice was added immediately after the 2-min oral digestion for evaluation of gastric digestion at time zero (G0). G0 was performed at 37 °C under constant stirring (75 rpm).

The pH levels were monitored before and after addition of digestive juices, and at the end of the digestive transit time. However, these parameters varied according to the seaweed species and harvesting mode (wild or cultivated). For this reason, after the addition of digestive juices, the pH was adjusted using 12 N HCl and 3 N NaOH solutions. For this adjustment, the ranges of 2.0 to 3.0 for gastric pH and 6.5 to 7.0 for duodenal pH were used [63]. At the end of the digestion process, digested seaweeds (pH adjusted to 7) were placed in 50 mL tubes and centrifuged for 20 min at 9800× *g* at 4 °C (Sorval Legend X1R, Thermo Fisher Scientific, Montreal, QC, Canada), yielding supernatant and pellet. Once the centrifugation of the duodenal digestion samples (D120) was completed, the enzymes were inactivated by treatment with protease inhibitors (Pefabloc SC). Samples were then frozen in liquid nitrogen and freeze-dried. Except for the addition of the inhibitor, the same procedure was applied to the G0 and G60 samples. The D120 freeze-dried samples were stored at −80 °C, and G0 and G60 at −20 °C until further use. A mixture (digestive juices + enzymes) without the seaweed sample (blank) was used simultaneously as a control for all digestive stages (G0, G60 and D120). The control (blank) was used to correct the values of protein release and undigested nutrients, serving to measure the contribution of digestive enzymes in the different measurements. The in vitro digestive process was performed in triplicate with repeated measurements for each step (including the control/blank) and seaweed sample.

A Micro N cube analyzer was used to determine the nitrogen content of all supernatants resulting from the digestive stages (G0, G60 and D120), using a nitrogen conversion factor of 4.92 [80]. Based on these data, total protein release concentration was calculated using Equation (1):

Protein release (%) = ((Protein content determined in supernatant (mg))/ (Protein content in seaweed before digestion (mg))) × 100,(1)

The degree of hydrolysis (DH) of each digestive supernatant (G0, G60 and D120) was determined by measuring the free amino groups using o-phthaldialdehyde (OPA), according to Church et al. and Nielsen et al. [81,82]. A standard curve was prepared with leucine at concentrations ranging from 0 to 12.3 mM. The DH was calculated as indicated by Bondu et al. [36]. 

### 4.4. Undigested Nutrients

Pellets resulting from centrifugation (G0, G60 and D120) were subject to fine grinding (model: Thermomix, Vorwerk, Wuppertal, Germany), and stored (−20 °C) in 50 mL tubes until further use. Pellets were dried (105 °C, 24 h) [83] and protein content was determined as described previously. Seaweed disintegration (D), representing the dispersion of seaweeds into the water phase during digestion (G0, G60 and D120), was calculated as shown in Equation (2):

D (%) = ((Initial weight of dry seaweeds before digestion − Weight of the dry pellets)/(Initial weight of dry seaweeds before digestion)) × 100(2)

Dry pellet samples of digestive stage D120 were separated and analyzed as for the soluble and insoluble fiber content determinations using the enzymatic-gravimetric method [77] to verify possible interference at the end of digestion. “Other nutrients” were calculated by difference as a function of the dry weight of the pellet, i.e., dry weight of the corrected pellet (without control/blank) subtracted from the dry weights of the protein, soluble and insoluble fibers.

### 4.5. Bioactivities

Product bioactivity measurements in the supernatant of duodenal digestion (D120) of wild and cultivated *P. palmata* and *S. latissima* samples were conducted using the Angiotensin-converting enzyme (ACE) inhibitory activity test and the oxygen radical absorbance capacity (ORAC) antioxidant assay, as described below. 

#### 4.5.1. ACE Inhibitory Activity

ACE inhibitory activity was measured by monitoring the formation of hippuric acid (HA) from the substrate hippuryl-L-histidyl-L-leucine (HHL) (at 12.5 mM) according to the method of Hayakari et al. and Suetsuna, with the modifications suggested by Bondu et al. [36,72,84]. This method is based on the colorimetric reaction between hippuric acid with 3% (v/v) 2,4,6-trichloro-s-triazine (TT) solution and 0.2 M potassium phosphate buffer (pH 8.3). Enalapril (10 mM) was used as a standard ACE inhibitor. The amount of HA formed during the reaction period was determined by spectrophotometry at 382 nm. The experiment was conducted in triplicate. The percentage of ACE inhibition was calculated from the Equation (3).

Inhibition activity (%) = [(Ec − Es)/(Ec − Eb)] × 100(3)

where Es is the absorbance of the reaction mixture (control), Ec is the absorbance of the buffer (test), and Eb is the absorbance when the stop solution was added before the reaction occurred (blank) [36]. 

#### 4.5.2. Antioxidant Capacity

The ORAC assay was carried out using a plate reader (Synergy™ HTX, Agilent, Santa Clara, CA, USA) with an adapted methodology [85,86,87,88,89]. Samples were extracted into acetone:water:acetic acid at a volume ratio of 70:29.5:0.5, then diluted in phosphate buffer. Diluted samples were put into a 96-well plate with fluorescein solution. The reaction was initiated by the addition of 2,2′-azobis(2-methylpropionamidine) dihydrochloride (AAPH) solution. Trolox was used as standard, so the results are expressed as µmol of Trolox equivalent (TE) per g of sample. The experiment was performed in triplicate.

### 4.6. Statistical Analysis

All data were expressed as mean ± standard deviation. A one-way ANOVA was used to compare means. Homoscedasticity was performed by graphical analysis of residuals. Normality verifications were performed using the Shapiro–Wilk and Anderson–Darling tests. All digestion data with repeated measures (protein release, disintegration percentage, and undigested nutrients) were analyzed using Fisher’s least significant difference (LSD) for pairwise comparisons of different treatment groups. Other statistical analyses (for water retention capacity, ACE inhibitory activity and ORAC) were performed using Tukey’s honest significance (HSD) multiple comparison of means. The General linear mixed model (GLMM) was used to verify the existence of a linear relationship between the responses and predictors (factors). The Pearson correlation coefficient (*r*) was used to test the relationships between the percentages (%) of disintegration, water-holding capacities, and undigested nutrients (protein, soluble and insoluble fibers, and other nutrients). A probability of 95% (*p* < 0.05) was used in all analyses. All tests were available in the SAS 9.1.3. software (SAS Institute Inc., Cary, NC, USA).

## 5. Conclusions

In conclusion, macroalgae samples were partially disintegrated (20–45%) after the proposed in vitro digestion model. *P. palmata* (wild and cultivated) exhibited slightly higher digestive efficiency than *S. latissima*, with superior values of protein release, degree of hydrolysis and disintegration for wild samples and lower content of undigested proteins than the latter. Cultivated samples did not show differences in digestibility level among species. Strongly associations detected between dissolution capacity of macroalgae (disintegration) and the rate of undigested proteins with the content of soluble and insoluble fibers in the seaweed samples indicated that the extent of disintegration at the gastric stage was higher in samples with a higher soluble fiber and smaller insoluble fiber contents. Regarding the bioactive properties of the studied macroalgae, wild *S. latissima* were the samples with the highest antioxidant activity (ORAC) with about 210 μmol TE g^−1^. This sample presented a high initial protein content, but was the one with lowest values of protein release and degree of hydrolysis after the gastrointestinal digestion, suggesting that other compounds in the sample contributed to the observed activity, such as phenolics. ACE inhibitory activity was highest (33%) for cultivated *P. palmata* samples, at 3 mg mL^−1^. Further elucidation of bioactivity mechanisms should consider a thorough characterization of the material, including that of compounds other than protein.

## Figures and Tables

**Table 1:** Fiber (soluble and insoluble) and protein contents of dry samples *P. palmata* and *S. latissima* as a function of the harvesting/origin of samples.

Species	Harvesting/ Origin	Soluble Fiber (%)	Insoluble Fiber (%)	Protein (%)
*P. palmata*	Wild	10.52 ± 0.05 ^a^	12.19 ± 0.05 ^d^	19.87 ± 0.85 ^a^
*P. palmata*	Cultivated	10.61 ± 0.15 ^a^	18.81 ± 0.08 ^b^	19.31 ± 0.62 ^a^
*S. latissima*	Wild	5.91 ± 0.64 ^b^	32.44 ± 0.12 ^a^	16.97 ± 0.20 ^b^
*S. latissima*	Cultivated	3.77 ± 0.09 ^c^	13.92 ± 0.02 ^c^	15.03 ± 1.23 ^c^

Values are presented as mean ± standard deviation (*n* = 3). Values from the same column with different letters are statistically different according to Tukey’s Studentized Range (HSD) at *p* < 0.05. Error bars represent standard deviations.

## Data Availability

The data presented in this study are available on request from the corresponding author.
